# Complete mitogenome of *Nemacheilus subfusca* (Cypriniformes: Cobitidae)

**DOI:** 10.1080/23802359.2016.1144111

**Published:** 2016-02-10

**Authors:** Chi Zhang, Bo Ma, Lei Li, Jianshe Zhou, Junhua Gong, Baohai Li

**Affiliations:** a Tibet Academy of Agricultural and Animal Husbandry Sciences, Lhasa, Tibet, People’s Republic of China;; b Ministry of Agriculture of China, Heilongjiang Fisheries Research Institute, Chinese Academy of Fishery Sciences, Haerbin, People’s Republic of China

**Keywords:** Complete, mitogenome, *Nemacheilus subfusca*, phylogenetic

## Abstract

The *Nemacheilus subfusca *(*N*. *subfusca*) is a species of the Noemacheilinae in the Cobitidae family, which has been few reported. The first complete mitochondrion of the *N*. *subfusca* is 16 585 bp in length, containing 13 protein-coding genes, two rRNA genes (12S and 16S rRNA), 22 tRNA genes and a control region (D-loop region). The longest protein-coding gene of this mitochondrion was *ND5* (1839 bp), and the 22 tRNA genes were range from 66 (*tRNA^Cys^*) to 76 (*tRNA^Lys^*) in length. The 12S rRNA (957 bp) and 16S rRNA (1677 bp) were separated by *Trna^Val^*, and the D-loop region located between *tRNA^Pro^* and *tRNA^Phe^* genes. The phylogenetic indicates that the *N*. *subfusca* was independent from other species of distinct genus in the *Noemacheilinae*, which provided the valuable evidence on phylogenetic relationship of the *N*. *subfusca* at the molecular level.

The Cypriniformes is the largest freshwater fish group in the world and had 2662 species in 1994 (Nelson 2006). As one family of Cypriniformes, the Cobitidae (23 genus and 200 species) have great morphological differences among numerous species, and distributed in Eurasia and nearby and parts of Africa (Sawada 1982). The *Nemacheilus subfusca* (Cypriniformes: Cobitidae) was found in Brahmaputra River in Tibet and India, and inhabited between the gravels and bank of rivers(Froese & Pauly 2000; Roskov et al. 2013). The sample and information resource of the *N*. *subfusca* are rare, and the taxonomic relation between *N*. *subfusca* and other species of the Cobitidae remains unknown. Identification of complete mitogenome of the *N*. *subfusca* would supplement the limited data on molecular level and be reference for systematics.

The *N*. *subfusca* was obtained from lower reach of Yarlung Zangbo River (Motuo, Tibet), and the fin was separated as a sample. The genomic DNA was extracted by the DNeasy Tissue Kit (Qiagen, Hilden, Germany), following the standard procedure. The complete mitochondrion sequence was amplified by PCR with distinct primers and assembled by DNAstar v7.1 (DNASTAR, Inc., Madison, WI). In general, the length of this sequence is 16 585 bp (GenBank accession no. KU380330), containing 13 protein-coding genes, two rRNA genes (12S and 16S rRNA), 22 tRNA genes and a control region (D-loop region), consisting of other mammals (Curole & Kocher 1999).

Among the protein-coding genes, 11 genes took the start codon of ATG, while *COX1* and *ND6* got GTG and CTA, respectively. The termination codon of these protein-coding genes had four types (six genes were TAA, three genes were TAG, three genes were “T– –” and one gene was CAT). The longest gene in this mitochondrion was *ND5* (1839 bp), and the 22 tRNA genes were range from 66 (*tRNA^Cys^*) to 76 (*tRNA^Lys^*) in length. The 12S rRNA (957 bp) and 16S rRNA (1677 bp) were separated by *Trna^Val^* gene, and the D-loop region located between *tRNA^Pro^* and *tRNA^Phe^* genes, with the length of 928 bp. Furthermore, three overlaps between protein-coding genes were found, including the *ATP6* and *ATP8* genes with 10 bp, *ND4L* and *ND4* genes with 7 bp, *ND5* and *ND6* genes with 4 bp, which consisted of *Beaufortia szechuanensis* (Wu et al. 2015).

The Cobitidae, one group of the *Cobitoidea*, had three subfamilies (Botiinae, Cobitidinae and Noemacheilinae). To explore the phylogenetic position and relationship of the *N*. *subfusca* among other species of the Noemacheilinae, the complete mitochondrion sequences of 11 species of three genus from the Noemacheilinae and the complete mitochondrion sequence of the *N*. *subfusca* in this study were used to construct the phylogenetic tree by MEGA6.06 software (MEGA Brands Inc., Montreal, QC) with ML analysis, and took the *Cobitis sinensis* and *misgurnus anguillicaudatus* as the outgroup (Figure 1). The neighbour-joining (NJ) tree (with 1000 bootstrap replicates) and the perfect bootstrap of each cluster verified that the *N*. *subfusca* was independent from Triplophysa (*Triplophysa anterodorsalis*, *Triplophysa bleekeri*, *Triplophysa stoliczkai*, *Triplophysa strauchii*, *Triplophysa robusta*, *Triplophysa siluroides* and *Triplophysa rosa*), Barbatula (*Barbatula barbatula*, *Barbatula nuda* and *Barbatula toni*) and Lefua (*Lefua echigonia*). This result provided the valuable evidence on phylogenetic relationship of the *N*. *subfusca* at the molecular level.

**Figure 1.**
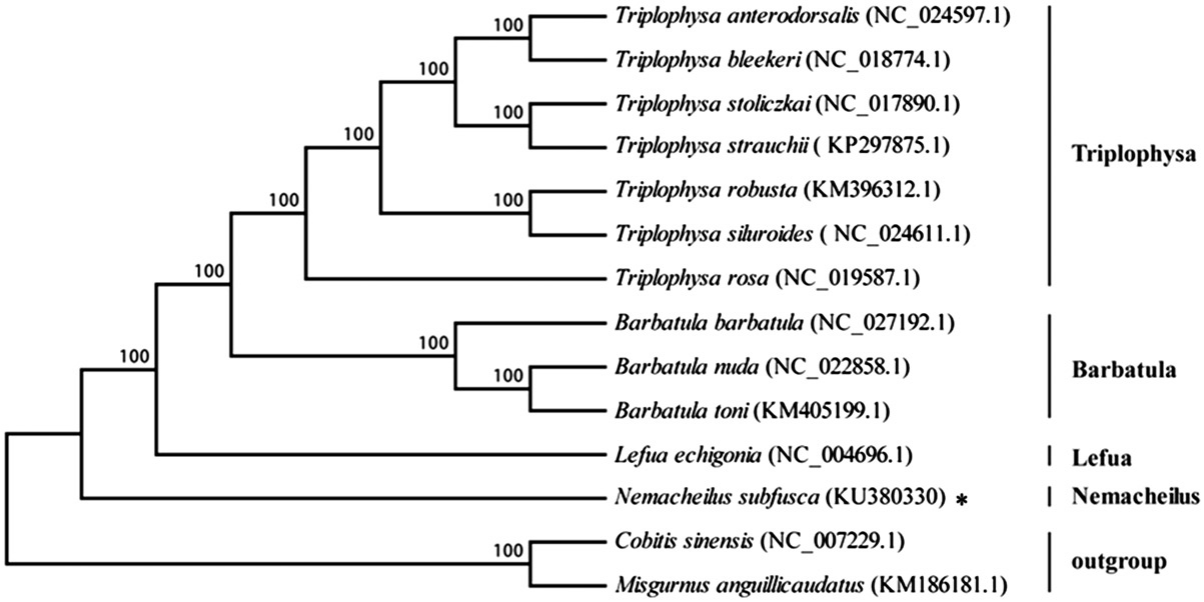
A neighbour-joining (NJ) tree of the *N. subfusca*. Two species of the Cobitinae (*Cobitis sinensis* and *Misgurnus anguillicaudatus*) were selected as outgroups. The numbers show the bootstrap value. The asterisk indicated the species in this study.
